# Duration differences of corticostriatal responses in striatal projection neurons depend on calcium activated potassium currents

**DOI:** 10.3389/fnsys.2013.00063

**Published:** 2013-10-04

**Authors:** Mario A. Arias-García, Dagoberto Tapia, Edén Flores-Barrera, Jesús E. Pérez-Ortega, José Bargas, Elvira Galarraga

**Affiliations:** Departamento de Neurociencia Cognitiva, División de Neurociencias, Instituto de Fisiología Celular, Universidad Nacional Autónoma de MéxicoMéxico City DF, México

**Keywords:** Ca^2+^-activated K^+^-currents, BK channels, SK channels, corticostriatal pathway, medium spiny neurons, striatum, synaptic integration

## Abstract

The firing of striatal projection neurons (SPNs) exhibits afterhyperpolarizing potentials (AHPs) that determine discharge frequency. They are in part generated by Ca^2+^-activated K^+^-currents involving BK and SK components. It has previously been shown that suprathreshold corticostriatal responses are more prolonged and evoke more action potentials in direct pathway SPNs (dSPNs) than in indirect pathway SPNs (iSPNs). In contrast, iSPNs generate dendritic autoregenerative responses. Using whole cell recordings in brain slices, we asked whether the participation of Ca^2+^-activated K^+^-currents plays a role in these responses. Secondly, we asked if these currents may explain some differences in synaptic integration between dSPNs and iSPNs. Neurons obtained from BAC D_1_ and D_2_ GFP mice were recorded. We used charybdotoxin and apamin to block BK and SK channels, respectively. Both antagonists increased the depolarization and delayed the repolarization of suprathreshold corticostriatal responses in both neuron classes. We also used NS 1619 and NS 309 (CyPPA), to enhance BK and SK channels, respectively. Current enhancers hyperpolarized and accelerated the repolarization of corticostriatal responses in both neuron classes. Nevertheless, these drugs made evident that the contribution of Ca^2+^-activated K^+^-currents was different in dSPNs as compared to iSPNs: in dSPNs their activation was slower as though calcium took a diffusion delay to activate them. In contrast, their activation was fast and then sustained in iSPNs as though calcium flux activates them at the moment of entry. The blockade of Ca^2+^-activated K^+^-currents made iSPNs to look as dSPNs. Conversely, their enhancement made dSPNs to look as iSPNs. It is concluded that Ca^2+^-activated K^+^-currents are a main intrinsic determinant causing the differences in synaptic integration between corticostriatal polysynaptic responses between dSPNs and iSPNs.

## Introduction

Ca^2+^-activated K^+^-currents are important regulators of excitability: they control firing frequency and synaptic integration (Bond et al., 2005; Salkoff et al., 2006; Faber, 2009). In striatal projection neurons (SPNs), Ca^2+^-activated K^+^-currents contribute to action potential repolarization and the afterhyperpolarization that makes up a great part of the interspike interval during repetitive firing (Pineda et al., 1992; Vilchis et al., 2000; Pérez-Garci et al., 2003; Pérez-Rosello et al., 2005; Wolf et al., 2005; Galarraga et al., 2007; Flores-Barrera et al., 2009). Selective peptidic toxins made clear that Ca^2+^-activated K^+^ currents in SPNs comprise “small” SK and “large” BK conductance channels (Pineda et al., 1992; Bargas et al., 1999). These channels are important targets for the actions of neurotransmitters, e.g.: dopamine, acetylcholine and somatostatin, among others (Pineda et al., 1995; Hernández-López et al., 1996; Vilchis et al., 2002; Pérez-Rosello et al., 2005; Galarraga et al., 2007). In dissociated neurons, Ca^2+^-activated K^+^-currents are preferentially activated by calcium entry through Ca_V_2.1 and Ca_V_2.2 channels. Ca_V_1 and Ca_V_2.3 calcium channel represent a much smaller Ca^2+^ source (Vilchis et al., 2000).

However, Ca^2+^ entry in the dendrites of SPNs is not mainly due Ca_V_2.1 and Ca_V_2.2 channels. Near synaptic sites of SPNs, NMDA, Ca_V_1, Ca_V_2.3, and Ca_V_3 channels become a main source of Ca^2+^ (Galarraga et al., 1997; Carter and Sabatini, 2004; Higley and Sabatini, 2008; Flores-Barrera et al., 2011). Because Ca^2+^-activated K^+^-channels could be present in the synaptic regions of dendrites (Ngo-Anh et al., 2005; Gu et al., 2008; Benhassine and Berger, 2009; Wynne et al., 2009; Faber, 2010; Grewe et al., 2010; Allen et al., 2011; Hosy et al., 2011; Tonini et al., 2013, e.g., Womack et al., 2004), one question to ask is whether Ca^2+^-activated K^+^ currents are involved in dendritic synaptic integration and the repolarization of polysynaptic corticostriatal responses (Vizcarra-Chacon et al., 2013), given that Ca^2+^ sources could be different than in the soma. Different sources of Ca^2+^ to activate Ca^2+^-dependent K^+^-currents in the dendrites and the soma-axon hillock regions could imply that synaptic inputs and the generation of action potentials may be regulated differentially. Thus, in the present work we describe the role of Ca^2+^-activated K^+^-currents in the corticostriatal responses and use them as reporters of the importance of Ca^2+^ influx into the dendrites during synaptic integration.

SK class channels are widely expressed in the brain and are found in dendritic spines near synaptic contacts (Ngo-Anh et al., 2005; Gu et al., 2008; Faber, 2010; Allen et al., 2011; Hosy et al., 2011; Tonini et al., 2013). BK class channels are functional in each neuronal compartment: soma, dendrites and terminals (Gu et al., 2008; Benhassine and Berger, 2009; Wynne et al., 2009; Grewe et al., 2010). Ca^2+^-activated K^+^ channels on neuronal dendrites can be activated by calcium influx through synaptic glutamate receptors and/or voltage-activated Ca^2+^-channels (Faber, 2009).

Because polysynaptic corticostriatal responses after a single stimulus are more prolonged, and evoke more action potentials, in direct pathway SPNs (dSPNs) than in indirect pathway SPNs (iSPNs) (Bargas et al., 1991; Flores-Barrera et al., 2010, 2011), a logical follow up of our previous studies had the following goals: first, identify whether Ca^2+^-activated K^+^-currents participate in the polysynaptic corticostriatal response of SPNs (Vizcarra-Chacon et al., 2013). Second, determine the relative importance of these currents in suprathreshold synaptic integration. Third, observe whether there is a difference in the roles of BK and SK currents in these responses. And finally, find out if these currents can explain the difference in duration between the corticostriatal polysynaptic responses of dSPNs and iSPNs.

## Materials and methods

### Slice preparation

All experiments were carried out in accordance with the National Institutes of Health Guide for Care and Use of Laboratory Animals and were approved by the Institutional Animal Care Committee of the Universidad Nacional Autónoma de México. D_1_ and D_2_ dopamine receptors-eGFP BAC transgenic mice, between postnatal days 30–60 (PD30-60; FVB background, developed by the GENSAT project) were used. Adult Wistar rats were also used to detect possible inconsistencies. The number of animals employed in the experimental samples was near the minimal possible to attain robust reproducible results and statistical significance. Animals were anesthetized with ketamine/xylazine. Their brains were quickly removed and placed into ice cold 4°C) cerebrospinal fluid (CSF) containing (in mM): 126 NaCl, 3 KCl, 25 NaHCO_3_, 1 MgCl_2_, 2 CaCl_2_, 11 glucose, 300 mOsm/L, pH = 7.4 with 95% O_2_ and 5% CO_2_. Parasagittal neostriatal slices (250–300 μm thick) were cut using a vibratome and stored in oxygenated bath CSF at room temperature for at least 1 h before recording.

### Electrophysiological recordings

Whole-cell patch-clamp recordings were performed with micropipettes made with borosilicate glass, fire polished for D.C. resistances of about 3–6 MΩ. Internal solution was (in mM): 120 KMeSO_4_, 2 MgCl_2_, 10 HEPES, 10 EGTA, 1 CaCl_2_, 0.2 Na_2_ATP, 0.2 Na_3_GTP, and 1% biocytin. Some recordings were carried out using sharp microelectrodes (80–120 MΩ) filled with 1% biocytin and 3M potassium acetate fabricated from borosilicate-glass (Flores-Barrera et al., 2010). Slices were superfused with CSF at 2 ml/min (34–36°C). Recordings were digitized and stored with the aid of software designed in the laboratory by one of the co-authors in the LabView environment (National Ins., Austin, TX, USA). To have an approximation of whole neuronal input resistance (R_N_) and changes in bridge balance, a small current pulse of −20 mV was delivered to the soma before the orthodromic responses were obtained at intervals over 20 s. Changes over 20% in the responses to this pulse or in the D.C. current to maintain the holding potential in current-clamp mode made us to discard the experiments. Sampling rate or digitizing procedures made difficult to have both the slow corticostriatal responses and full-blown trains of sodium spikes at the same time, so that some spikes look truncated in some traces. Cells with resting potential more negative than −70 mV (at zero current) and R_N_ about 100–200 MΩ were chosen (e.g., Salgado et al., 2005).

Drugs were dissolved in the CSF from stock solutions made daily. Recordings were carried out in the dorsal striatum. Stimulation was performed with concentric bipolar electrodes (tip = 50 μm) in the cortex (>250 μm from the callosum). Synaptic responses were evoked by a single square pulse of 0.1 ms. The cell membrane potential was held at −80 mV. A series of current pulses of increasing intensities were used. Responses obtained with suprathreshold stimulus strength (2× threshold) were compared (Vizcarra-Chacon et al., 2013). Current-clamp data were obtained to observe the most physiological response. eGFP-positive and negative neurons from D_1_ and D_2_ eGFP animals were compared. After recordings, neurons were injected with biocytin. eGFP-positive visualization was observed on a confocal microscope as previously described (Flores-Barrera et al., 2010).

The following drugs: apamin and charybdotoxin were obtained from Alomone Labs (Israel). NS 1619 (BK channel activator), CyPPA, and NS 309 (SK channel activators) were acquired from TOCRIS (R&D Systems, Minneapolis, MN, USA).

### Statistics

Statistical values are presented as mean ± s.e.m. Digital subtraction was used to obtain time courses of the components enhanced or decreased by the different peptides and drugs. Main parameter reported was “half width” (duration at 50% of maximal response amplitude) because it comprises both the increase in response duration and the increase in response amplitude. Kruskal-Wallis tests with *post-hoc* Dunn tests were used to compare samples with different treatments. Statistical difference was accepted when *P* < 0.05. Intensity-response (I-R) plots where built using as response the average magnitude of the half width as a function of stimulus strength. Data were fitted to the following sigmoid function using a non-linear Marquardt algorithm:

$$R(i)=\frac{R_{\max}}{1+e^{-k(i\;-\;ih)}}$$

where *R*(*i*) is the response as a function of stimulus intensity normalized to threshold units, *R*_max_ is the maximal half width attained, *k* is the slope factor and *ih* is the stimulus intensity necessary to attain a response of half magnitude. In this work, the value reported is *R*_max_. The function fits to average experimental values and their 95% confidence intervals are plotted as colored bands (Tecuapetla et al., 2005; López-Huerta et al., 2013) while standard errors of average responses are plotted as usual: lines with the symbols.

## Results

SPNs were recorded from PD30-60 BAC D_1_ or D_2_ eGFP mice and from rats of equivalent age (*n* = 77). We previously reported that suprathreshold corticostriatal responses are more prolonged and evoke more action potentials in dSPNs than in iSPNs (Flores-Barrera et al., 2010) and that their duration depends on polysynaptic activity (Vizcarra-Chacon et al., 2013) involving both cortical and striatal neurons (a feed-forward circuit) as well as intrinsic currents (Vergara et al., 2003; Flores-Barrera et al., 2009, 2011).

### Blockade of BK and SK channels depolarize and prolong corticostriatal responses in striatal projection neurons

**Figure 1:**
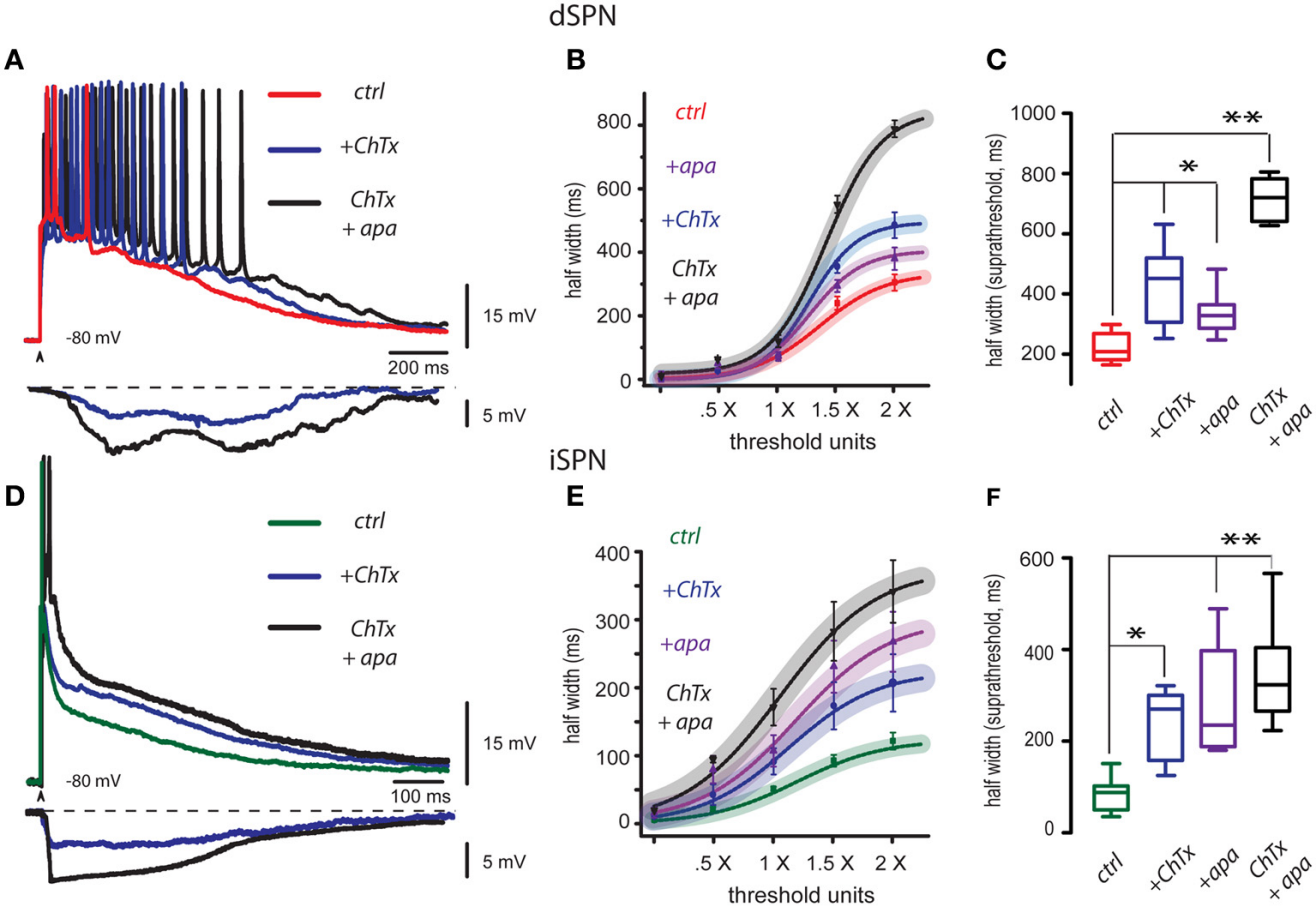
**Blockade of BK and SK channels depolarize and prolong corticostriatal responses in striatal projection neurons. (A)** Three superimposed records from a dSPN were obtained without changing stimulus strength: they show a corticostriatal response in control conditions (red trace), the same response in the presence of the blocker of BK-channels, 20 nM charybdotoxin (ChTx, blue trace), and finally, the same response in the presence of both ChTx and the blocker of SK channels, 100 nM apamin (ChTx + apamin, black trace). Each blocker depolarized and prolonged the response, and also increased the number of action potentials that were fired. Digital subtractions at bottom illustrate the hyperpolarizing influence that Ca^2+^-activated K^+^-currents exerted over the corticostriatal responses and that were suppressed by the blockade of ChTx (blue) and apamin (black). Note that the hyperpolarizing influence of Ca^2+^-activated K^+^-currents rise slowly and last hundreds of milliseconds thus contributing to restrain the build up of the corticostriatal response. **(B)** Intensity-response (I-R) graphs obtained by plotting average half width (duration at 50% of the peak amplitude of the response) as a function of threshold intensity including: minimal, subthreshold (0.5×), threshold (1.0×) and suprathreshold responses (2.0×). A sigmoid function was fitted. Curves with the action of each blocker are plotted individually as well as the administration of both blockers together. Shadowed colored areas denote 95% confidence intervals, symbols depict average values of the samples for each stimulus strength ± s.e.m. **(C)** Tukey box plots illustrate the distributions of measurements for suprathreshold responses (half width at 2× threshold strength), in control, in the presence of each blocker, and in the presence of both blockers. Differences are significant: ^*^P < 0.5 and ^**^P < 0.01 with respect to the controls. **(D)** Three superimposed records from an iSPN were obtained without changing stimulus strength: they show a corticostriatal response in control conditions (green trace), the same response in the presence of the blocker of BK channels, 20 nM charybdotoxin (ChTx, blue trace), and finally, the same response in the presence of both ChTx and apamin (ChTx plus apamin, black trace). As in the case of dSPNs, each blocker increased response duration and depolarization (half width). Subtractions at the bottom show the hyperpolarizing influences exerted by Ca^2+^-activated K^+^-currents that were suppressed by charybdotoxin (ChTx, blue trace) and apamin plus ChTx (black trace). Note that hyperpolarization induced by Ca^2+^-activated K^+^-currents rise fast and then produced a sustained plateau hyperpolarization that restrains the corticostriatal response by hundreds of milliseconds until final return to holding potential. **(E)** Fitted I-R plots (half width) were graphed individually for each blocker and for both blockers administered together as in **(B)**. **(F)** Box plots summarize the distributions of measurements for suprathreshold response in control, with each blocker, and with both blockers administered together. Differences are significant: ^*^P < 0.5 and ^**^P < 0.01 with respect to the control. Due to the intent of showing the slow responses some fast spikes are truncated by the digitizing procedure.

Red trace in Figure 1A is a control corticostriatal response in a dSPN. The superimposed blue trace is a response obtained with the same stimulus and in the same cell after adding 20 nM charybdotoxin (ChTx), a blocker of BK-channels, to the bath CSF: the response exhibited an additional depolarization that prolonged the response and the number of spikes. The superimposed black trace is the corticostriatal response to the same stimulus after adding 100 nM apamin, a blocker of SK-channels, in the continuous presence of ChTx. This response was even more depolarized, more prolonged and the number of action potentials fired increased, showing that Ca^2+^-activated K^+^-currents control the level and duration of corticostriatal responses which physiologically compose the depolarized up-states (Stern et al., 1997; Vergara et al., 2003; Vautrelle et al., 2009) of SPNs. At the bottom, digital subtractions of the hyperpolarizing influences elicited by Ca^2+^-activated K^+^-currents that were suppressed by the actions of these peptides are illustrated (ChTx in blue and apamin plus ChTx in black). Note that hyperpolarizations induced by Ca^2+^-activated K^+^-currents rise slowly and lasts hundreds of milliseconds, suggesting that, after synaptic activation, Ca-entry takes some time to activate BK- and SK-channels in dSPNs. The parameter that encompasses both the depolarization and the increase in duration produced by the peptides is the half width of the response (duration at half amplitude). Figure 1B shows that half width as a function of stimulus intensity (in threshold units) could be used to build I-R plots, which could be fitted to sigmoid functions (see Materials and Methods): continuous traces represent the average fit of several similar experiments and surrounding shadowed areas represent 95% confidence intervals. Symbols with lines represent individual average responses ± s.e.m. to each stimulus. Note that peptides, administered jointly, or independently, change I-R plots with respect to the control, although all plots tend to reach saturation. Box plots in Figure 1C summarize the actions of both peptides in the experimental samples (because not all distributions approached normality, free-distribution statistical tests were used for comparisons; see Materials and Methods), including a sample where both peptides were applied together in either order at 2× threshold strength (saturation). Half width in the control was (mean ± s.e.m.): 296 ± 26 ms (*n* = 24, in Red), after ChTx it was 474 ± 40 ms or 60% increase (*n* = 10; in blue, ^*^P < 0.05), after apamin it was 370 ± 53 ms or an increase of 25% (in purple, *n* = 10; ^*^P < 0.05), and with both peptides applied together it was 773 ± 26 ms or a 161% increase (in black, *n* = 6; ^**^P < 0.02). Fitted values of the same parameter (e.g., *R*_max_ for maximal half width; see Materials and Methods) were not significantly different to experimental measurements (Figure 1B) and therefore are not included here to avoid redundancy.

The same experiments were performed in iSPNs. Half width of iSPNs is known to be briefer than that from dSPNs (Flores-Barrera et al., 2010). These neurons commonly fire a single or brief high frequency burst of spikes at the beginning of the synaptic response. As above, Figure 1D shows three superimposed records: the green one was taken in control conditions, the blue trace was obtained with the same stimulus and in the same cell after addition of 20 nM charybdotoxin (+ChTx) and the black trace was obtained after addition of 100 nM apamin (+apa) in the continuous presence of charybdotoxin. Each peptide depolarized and prolonged the response (half width). At the bottom, subtractions of the hyperpolarizing influences elicited by Ca^2+^-activated K^+^-currents blocked by these peptides are illustrated (charybdotoxin in blue and ChTx plus apamin in black). Note that hyperpolarizions induced by Ca^2+^-activated K^+^-currents in iSPNs show a major difference when compared to those obtained from dSPNs: hyperpolarizations begin quickly, and then are followed by a plateau-like hyperpolarization that restrains the corticostriatal response by hundreds of milliseconds to slowly return to holding potential during the last half of the response. These responses suggest that in contrast to dSPNs, Ca-entry activates BK- and SK-channels immediately at the beginning of the synaptic response in iSPNs, suggesting that Ca-entry that induces them probably comes from all-or-none dendritic phenomena that produce the Ca-influx (Bargas et al., 1991; Flores-Barrera et al., 2010; Higley and Sabatini, 2010; Plotkin et al., 2011; Vizcarra-Chacon et al., 2013). Figure 1E shows fitted I-R plots for each peptide acting independently or together in either order. Continuous traces are the function fits for the set of experiments and surrounding shadowed correspond to 95% confidence intervals. Symbols with lines are average measurements ± s.e.m. Box plots in Figure 1F summarize the actions of both peptides in different samples, including the sample where both peptides were applied together in either order: at 2 × threshold strength, the control was 115 ± 13 ms (in green, *n* = 19), while after ChTx it was 204 ± 44 ms or a 77% increase (in blue, *n* = 7; ^*^P < 0.05), and after apamin it was 267 ± 46 ms or a 132% increase (in purple, *n* = 8; ^**^P < 0.02). Both peptides applied together rendered a half width of 344 ± 48 ms or a 199% increase (in black, *n* = 6; ^**^P < 0.02).

### Enhancement of BK and SK channels hyperpolarize and shorten corticostriatal responses in striatal projection neurons

**Figure 2:**
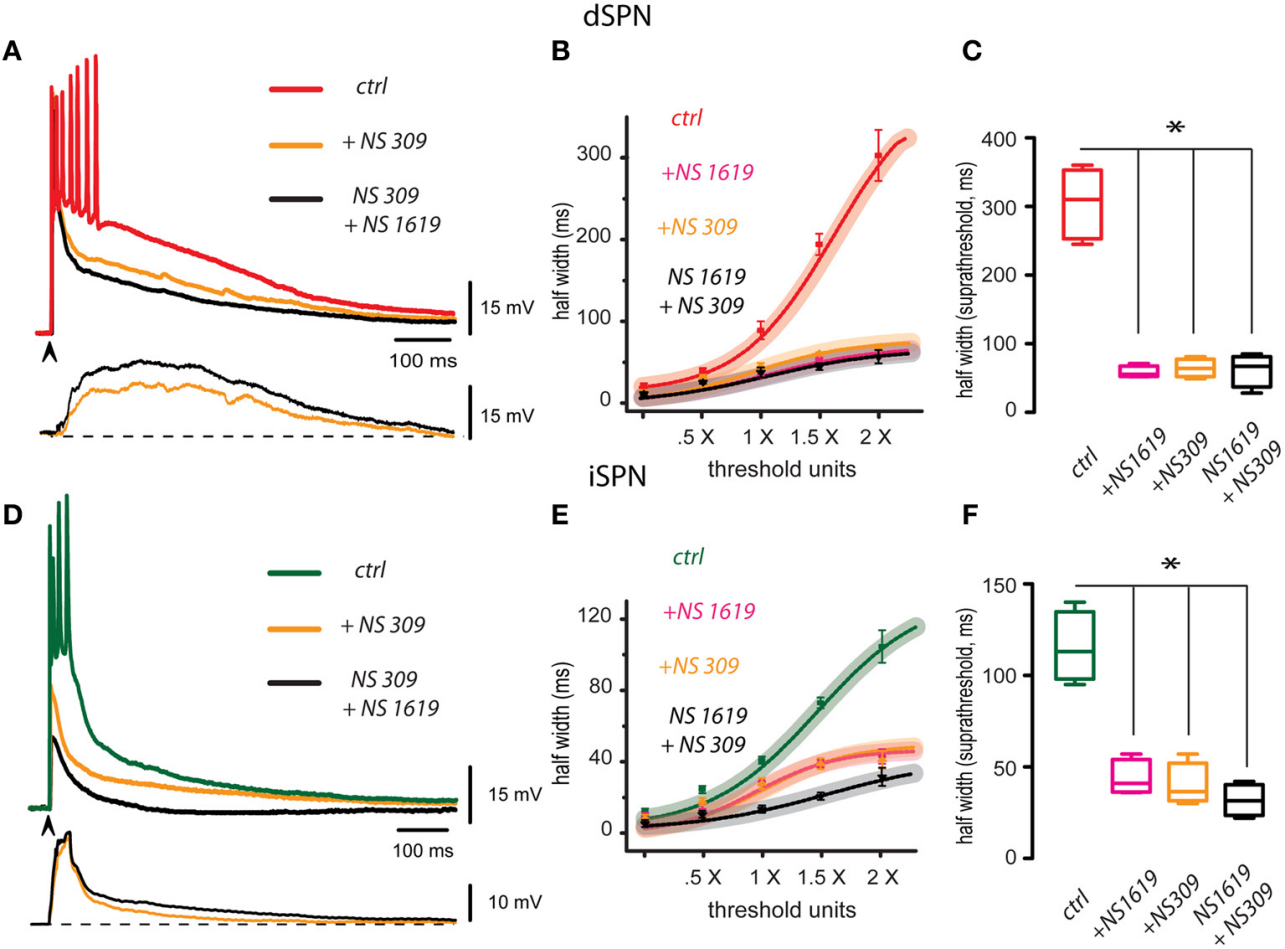
**Enhancement of BK and SK channels hyperpolarizes and shortens corticostriatal responses in striatal projection neurons. (A)** Three representative records from a dSPN were obtained in the same cell without changing stimulus strength (superimposed): the red trace shows a corticostriatal response in control conditions, the same response is shown in the presence of 2.5 μM NS 309 (orange trace), an activator of SK-channels, and finally, the same response is shown in the presence of both NS 309 and the activator of BK-channels, 2.5 μM NS 1619 (black trace). Each activator hyperpolarized the response, shortened its duration, and decreased the number of action potentials fired. Subtractions of the response depolarizations blocked by NS 309 (orange) and NS 309 plus NS 1619 (black) are shown at the bottom. Note that depolarizations blocked by these drugs begin slowly and last hundreds of milliseconds. Therefore, their blockade greatly reduced the suprathreshold responses **(B)**. Fitted I-R plots with 95% confidence intervals and experimental averages ± s.e.m. Action of each drug is plotted individually as well as the administration of both activators together. **(C)** Box plots summarize the distribution of measurements for suprathreshold response (half width at 2×) in different samples: control, with each activator, and with both activators administered together. Samples differed significantly from the control (^*^P < 0.05). **(D)** Three superimposed records from an iSPN were obtained without changing stimulus strength: they show a corticostriatal response in control conditions (green trace), the same response in the presence of the activators of SK channels, 2.5 μM NS309 (orange trace), and finally, the same response in the presence of both NS309 and 2.5μM NS1619 (black trace). As in the case of dSPNs, each blocker decreased response duration and depolarization (half width). Subtractions of the depolarizations blocked by NS309 (orange) and NS309 plus NS1619 (black) are shown at the bottom. Note that depolarizations in this case had a sudden initiation and repolarization. **(E)** Intensity-response (I-R) graphs obtained by plotting half width (duration) at 50% of the peak amplitude of the response as a function of threshold intensity including subthreshold (0.5×), threshold (1.0×), and suprathreshold responses (2.0×). Action of each activator is plotted individually as well as the administration of both drugs together. **(F)** Box plots summarize the distribution of samples for suprathreshold response (half width at 2×) in control, with each activator, and with both drugs administered together. Differences were significant (^*^P < 0.05). Although some fast spikes are truncated by the digitizing procedure, note that in these cases some initial spikes exhibit inactivation due to their relative refractory periods.

Red trace in Figure 2A is a control corticostriatal response in a dSPN. The superimposed orange trace is the same response obtained with the same stimulus and in the same cell after adding 2.5 μM NS 309, an activator of SK-channels, to the bath CSF: response depolarization decreased and duration became briefer. 2.5 μM CyPPA had the same actions (not illustrated). Finally, the same response is shown in the presence of both NS 309 and the activator of BK-channels: 2.5 μM NS 1619 (black trace): there was a larger reduction of the response, repolarization was faster and number of action potentials fired was reduced to one. These actions show that a positive modulation of Ca^2+^-activated K^+^-currents may control the magnitude of the corticostriatal responses in dSPNs. At the bottom, subtractions illustrate the cortical-induced depolarizations suppressed by these drugs (NS 309 in orange and NS 1619 plus NS 309 in black). That is, enhancement of Ca^2+^-activated K^+^-currents may suppress a great part of the prolonged depolarizing corticostriatal response. Nonetheless, note that these depolarizing components begin and end slowly in dSPNs. Figure 2B shows that I-R plots produced by any enhancer or a combination of them were depressed with respect to the control. Box plots in Figure 2C summarize the actions of both enhancers in different experimental samples, including a sample where both drugs were applied together in either order at 2× threshold strength. Half width in the control was (mean ± s.e.m.): 302 ± 31 (*n* = 18, in red), after NS 1619 it was 57 ± 2 ms or a 81% decrease (*n* = 6; in pink, ^*^P < 0.05), after NS 309 it was 65 ± 6 ms or a 78% decrease (in orange, *n* = 6; ^*^P < 0.05), and with both activators applied together it was 54 ± 16 ms or a 82% decrease (in black, *n* = 5; ^*^P < 0.05).

Green trace in Figure 2D is a control corticostriatal response in an iSPN. The superimposed orange trace is the same response after adding 2.5 μM NS 309 (SK-channels enhancer). Note that response depolarization decreased, duration became briefer and less action potentials were fired. 2.5 μM CyPPA had the same actions (not illustrated). Also superimposed, the response of the same cell is shown in the presence of both NS 309 and 2.5 μM NS 1619 (black trace). Note that cell response was reduced to the point of becoming subthreshold. These actions show that Ca^2+^-activated K^+^-currents may modulate corticostriatal responses in iSPNs. At the bottom, subtractions showing the depolarizing components of the corticostriatal responses shunted by the enhancement of Ca^2+^-activated K^+^-currents are illustrated (NS 309 in orange and NS 1619 plus NS 309 in black). Note that actions of these drugs begin and return faster, as compared to those from dSPNs. Figure 2E shows that I-R plots produced by any enhancer or a combination of them were depressed with respect to the control. Box plots in Figure 2F summarize the actions of both enhancers in the experimental samples, including a sample where both drugs were applied together in either order at 2× threshold strength. Half width in the control was (mean ± s.e.m.): 110 ± 9 ms (*n* = 10, in green), after NS 1619 it was 44 ± 4 ms or a 60% decrease (*n* = 6; in pink, ^*^P < 0.05), after NS 309 it was 40 ± 5 ms or a 62% decrease (in orange, *n* = 6; ^*^P < 0.05), and with both activators applied together it was 38 ± 16 ms or a 64% decrease (in black, *n* = 4; ^*^P < 0.05).

To conclude, in both classes of SPNs Ca^2+^-activated K^+^-currents control the level of depolarization and duration of the prolonged polysynaptic corticostriatal response during synaptic integration. These results strongly suggest that intrinsic currents such as Ca^2+^-activated K^+^-currents may control the influence of polysynaptic entries and their consequent activation of intrinsic currents contributing to up-states duration. However, a main difference between both classes of SPNs is that in iSPNs, a much larger influence of Ca^2+^-activated K^+^-currents appears to be present, triggered at the initiation of the synaptic inputs and immediately as in iSPNs. Next, we studied the influence of Ca^2+^-activated K^+^-currents in subthreshold synaptic responses, before action potentials were elicited, to see if there is a difference between both neurons classes.

### Ca^2+^-activated K^+^-currents control subthreshold synaptic inputs in iSPNs

**Figure 3:**
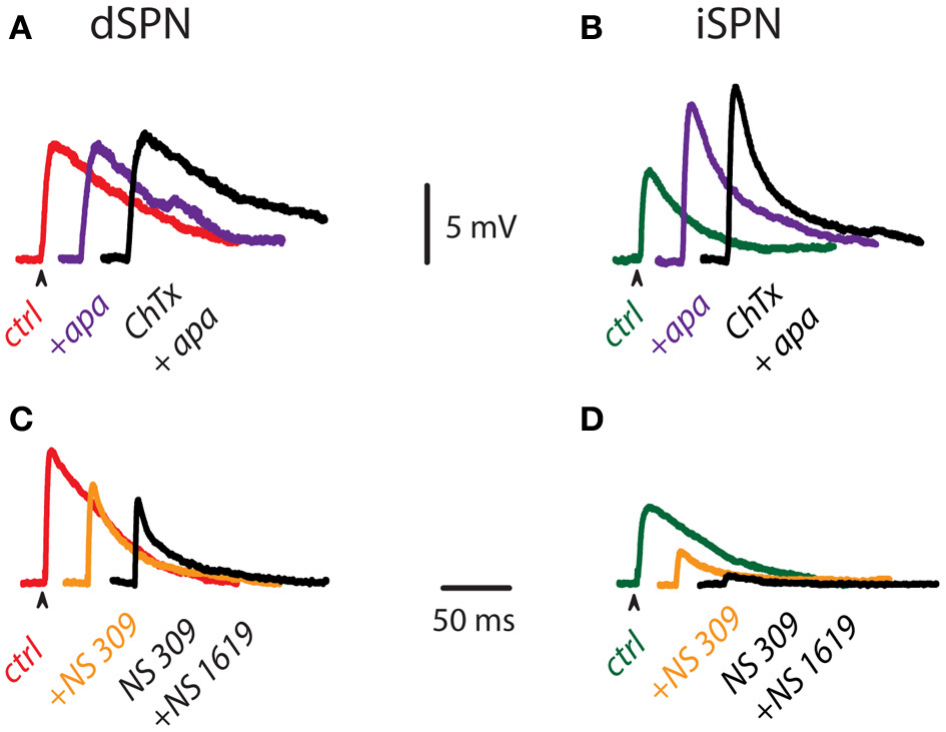
**Influence of Ca^2+^-activated K^+^-currents on subthreshold corticostriatal responses. (A)** Subthreshold synaptic potentials recorded after cortical stimulation in a dSPN in control conditions (red), after addition of 100 nM apamin (purple) and after addition of 20 nM ChTx in the continuous presence of apamin (black). **(B)** The same experiment was performed on an iSPN (control condition is in green). Note that Ca^2+^-activated K^+^-currents appear to exert more influence on subthreshold synaptic events of iSPNs. **(C)** Subthreshold synaptic potentials recorded after cortical stimulation in a dSPN in control conditions (red), after addition of 2.5 μM NS 309 (orange), an enhancer of SK-channels, and after addition of 2.5 μM NS 1619, an enhancer of BK-channels, in the continuous presence of NS 309 (black). **(D)** The same experiment performed on an iSPN (control condition is in green). Enhancers of Ca^2+^-activated K^+^-currents also indicate more influence of these currents in subthreshold synaptic events of iSPNs.

Figures 3A,B illustrate subthreshold synaptic potentials recorded after cortical stimulation in a dSPN and an iSPN in three different conditions: control (red and green traces for dSPN and iSPN, respectively), after addition of 100 nM apamin to block SK-channels (purple trace), and after addition of 20 nM ChTx in the continuous presence of apamin to block both SK- and BK-components of Ca^2+^-activated K^+^-currents (black traces). Note that in the case of dSPNs, calcium influx during subthreshold synaptic events does not appear to activate much Ca^2+^-activated K^+^-currents since the subthreshold synaptic potentials are very little affected. In comparison, blockade of Ca^2+^-activated K^+^-currents in iSPNs greatly alters the amplitude of subthreshold synaptic events, suggesting that calcium-influx capable to activate these currents can be generated with a minimum of active synapses (Higley and Sabatini, 2010). Because these events most probably occur in dendritic spines, we conclude that calcium influx during subthreshold synaptic events is enough to activate Ca^2+^-activated K^+^-currents in iSPNs, but not in dSPNs. Figures 3C,D show similar experiments with the enhancers of Ca^2+^-activated K^+^-currents. First, subthreshold synaptic potentials were recorded in control conditions (red and green traces for dSPN and iSPN, respectively), then the same events were recorded during enhancement of SK-channels (orange traces, NS 309), and finally, the same events were recorded during enhancement of both SK- and BK-current components (black traces, with NS 309 plus NS 1619). Enhancers of Ca^2+^-activated K^+^-currents do affect the amplitude of subthreshold synaptic potentials in dSPNs, showing that, if activated by some other means (more or larger synaptic potentials or spatial summation, as in suprathreshold responses), the channels that carry these currents are near enough to shunt synaptic inputs. However, enhancers of Ca^2+^-activated K^+^-currents reduce subthreshold synaptic potentials induced in iSPNs in such an extent as to almost completely block the synaptic event. These results suggest that in iSPNs, synaptic inputs may activate Ca^2+^-activated K^+^-currents augmenting the action of the enhancers and exerting a much larger effect as compared to dSPNs.

We conclude that even at subthreshold levels, Ca-entry during synaptic potentials in iSPNs is powerful enough to activate Ca^2+^-activated K^+^-currents and then shunt synaptic entries, to a degree that, during suprathreshold polysynaptic events (Vizcarra-Chacon et al., 2013) their duration and depolarization (half width) are greatly reduced in comparison to the same events generated in dSPNs. To further support these conclusions, we tried to control the corticostriatal suprathreshold responses in these neurons by manipulating their Ca^2+^-activated K^+^-currents.

### Controlling voltage trajectories of corticostriatal responses

**Figure 4:**
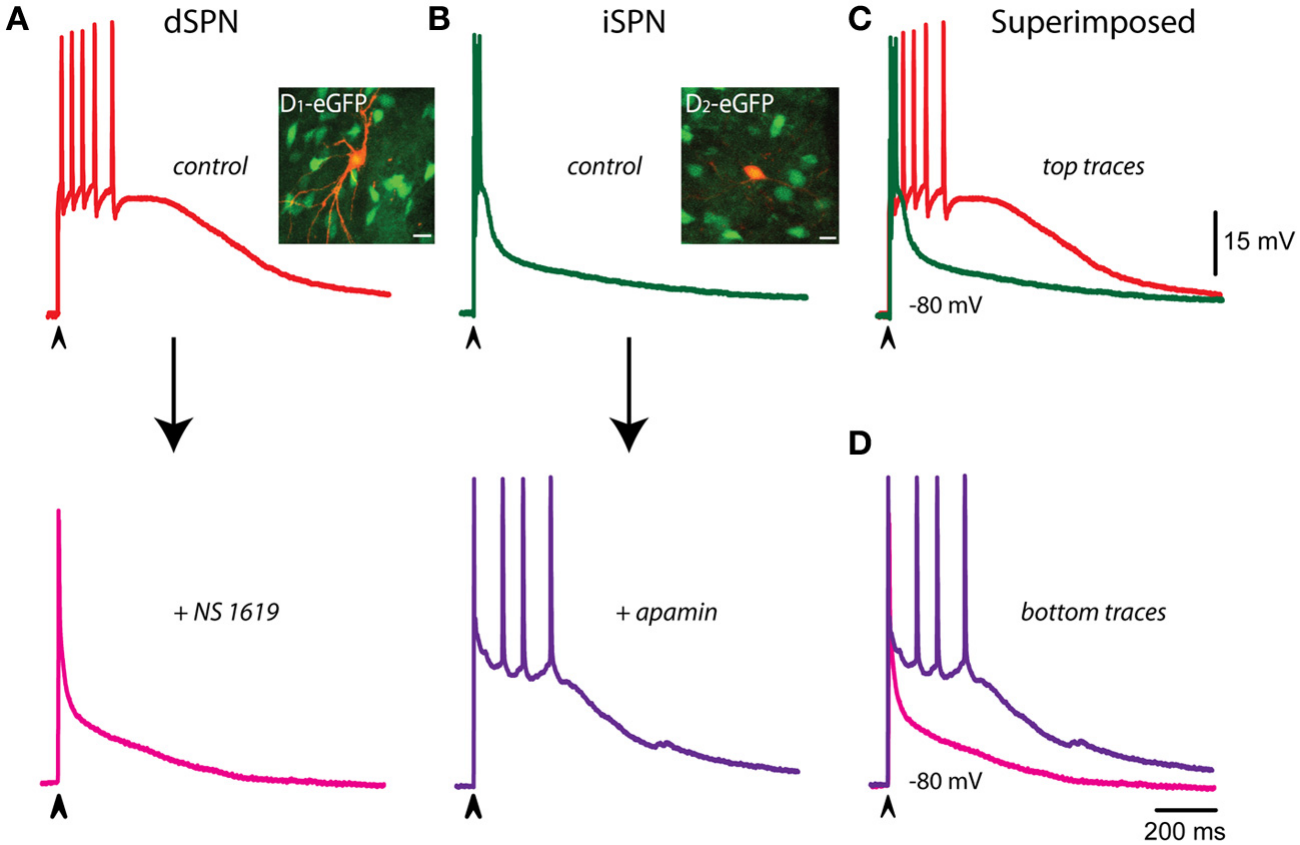
**Controlling voltage trajectories of corticostriatal responses: converting responses from dSPNs or iSPNs into one another. (A)** Control corticostriatal response in a dSPN (top). The arrow indicates the addition of 2.5 μM of the of the BK-channel activator NS 1619. Note that repolarization was enhanced and half width decreased, the corticostriatal response becoming comparable to that of an iSPN in control conditions (bottom). Inset: immunocytochemical preparation showing neurons from a BAC-D_1_ eGFP mouse (green) and the recorded neuron filled with biocytin (orange). **(B)** Control corticostriatal response in an iSPN. The arrow indicates the addition of 100 nM of the SK-channel blocker apamin. Note that fast repolarization and the firing of a brief burst of action potentials at the beginning of the response were changed by a response with increased duration (half width) and a more prolonged train of action potentials firing at lower frequency; comparable to those seen in control dSPNs. Inset: immunocytochemical preparation showing neurons from a BAC-D_2_ eGFP mouse (green) and the recorded neuron filled with biocytin (orange). **(C)** Superimposition of control dSPNs and iSPNs responses clearly shows larger half width for dSPNs. **(D)** After the actions of NS 1619 and apamin in dSPNs and iSPNs, respectively, half width of iSPNs became larger than that of a dSPNs, strongly suggesting that the shape of the corticostriatal responses in these neurons depend on Ca^2+^-activated K^+^-currents. Experiment made by triplicate and with different blockers and enhancers. Although the digitizing procedure may show incomplete fast spikes, the increase in the amplitude of some spikes in the dSPN recording are due to longer interspike intervals (**A**, top). Note constancy of spikes amplitude in **(B)**, bottom.

The question underlying this series of experiments is: what are the main determinants of the different voltage trajectories found in dSPNs, as compared with iSPNs, during corticostriatal responses? Figure 4A shows that when the corticostriatal response from a dSPN is subject to an enhancer of a Ca^2+^-activated K^+^-current (BK-channels component enhanced by NS 1619 in this case), the response acquires a faster repolarization, shortens its train of spikes and becomes similar to a typical corticostriatal response from an iSPN. This result suggests that the reason why dSPNs responses are slower than those from iSPNs is a decreased activation of Ca^2+^-activated K^+^-currents. Conversely, Figure 4B shows that when the corticostriatal response from an iSPN, exhibiting a brief train of spikes and a faster repolarization, is subject to a blocker a Ca^2+^-activated K^+^-current (SK-channels component blocked by apamin in this case), the response is prolonged, its repolarization retarded and its train of spikes increases in duration, becoming similar to a corticostriatal response from a dSPN. This result support the inference that the reason why iSPN responses are faster than those from dSPNs is an increased activation of Ca^2+^-activated K^+^-currents. Superimposition of the traces in each condition supports these hypotheses (Figures 4C,D).

### Ca^2+^-activated K^+^-currents and propagation of dendritic autorregenerative events in iSPNs

It has been shown that the reason why iSPNs fire a brief train of action potentials at the beginning of the corticostriatal response and then repolarize faster than dSPNs, is the presence of autoregenerative events in their dendrites: the brief train of action potentials rides on top of autoregenerative events that are elicited as local dendritic responses (Carter and Sabatini, 2004; Day et al., 2008; Flores-Barrera et al., 2010). However, in some occasions dendritic autoregenerative evens propagate to the somatic compartment inactivating the brief burst of spikes (Flores-Barrera et al., 2010). It is out of the scope of the present report to find out what is the origin and ionic constitution of these regenerative events in iSPNs (but see: Higley and Sabatini, 2010). However, several independent reports using different methods have posited that different classes of voltage-activated calcium channels as well as NMDA channels may be the origin of these events (Sabatini and Svoboda, 2000; Sabatini et al., 2002; Carter and Sabatini, 2004; Carter et al., 2007; Higley and Sabatini, 2008; Flores-Barrera et al., 2009; Plotkin et al., 2011). Moreover, channels that carry Ca^2+^-activated K^+^-currents have been shown to be present in dendritic spines or nearby dendrites in many neuron classes (Wolfart and Roeper, 2002; Cai et al., 2004; Bond et al., 2005; Ngo-Anh et al., 2005; Gu et al., 2008; Benhassine and Berger, 2009; Faber, 2009, 2010; Lujan et al., 2009; Hopf et al., 2010; Allen et al., 2011; Hosy et al., 2011), and data from striatal neurons is very suggestive that, at least in iSPNs, a tight synaptic association between synaptic, voltage-activated Ca^2+^-channels and Ca^2+^-activated K^+^-channels may be present (Day et al., 2008; Higley and Sabatini, 2008; Hopf et al., 2010).

**Figure 5:**
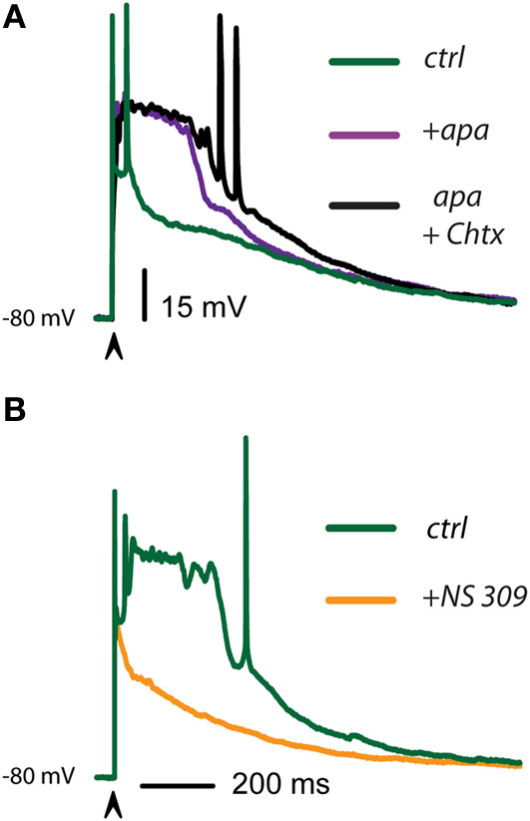
**Ca^2+^-activated K^+^-currents control the propagation of autoregenerative potentials in iSPNs. (A)** Green trace shows a control suprathreshold corticostriatal response in an iSPN. Addition of 100 nM apamin discloses an autoregenerative and propagated action potential whose main ionic component is Ca^2+^ (purple trace; Bargas et al., 1991). Subsequent addition of 20 nM ChTx in the continuous presence of apamin further prolongs the duration of the regenerative event without increasing its amplitude, illustrating its all-or-none properties (black trace). **(B)** Green trace is a spontaneous regenerative event that sometimes appears in control suprathreshold responses in iSPNs (alternating with the initial burst of spikes). Addition of 2.5 μM NS 309 hindered the propagation of this event to the somatic area and accelerated the repolarization (orange trace). Clearly, fast spikes inactivate when calcium autoregenerative potentials propagate.

The above described phenomena suggest that Ca^2+^-activated K^+^-currents may control autoregenerative events initiated by dendritic synaptic inputs, thus interfering with their propagation to the somatic-axonal compartments at most times. An experiment showing that this inference may be true is shown in Figure 5A: the green trace shows a couple of actions potentials riding on top of a suspected dendritic regenerative event taking place during synaptic activation. The subsequent addition of 100 nM apamin discloses the regenerative event by promoting its propagation to the soma (purple trace). Further addition of 20 nM ChTx in the continuous presence of apamin (black trace) makes this event more prolonged and capable to induce the firing of fast spikes. These results suggest that Ca^2+^-activated K^+^-currents were preventing autoregenerative events in the dendrites to propagate to the somatic compartment. In addition, in iSPNs, autoregenerative events sometimes take the place instead of the initial bursts of sodium spikes (Flores-Barrera et al., 2010). Green trace in Figure 5B illustrates one such a case. It is also shown that addition of NS 309 (orange trace) rapidly abolished the propagation of the regenerative event producing a fast repolarization. These experiments suggest that Ca^2+^-activated K^+^-currents control synaptic integration in SPNs, in particular, they avoid propagation of regenerative events to the somatic area, secluding them within dendritic compartments. Their propagation would make inefficient the generation and propagation of a burst of action potentials out to the axon.

## Discussion

Briefly, the present work shows that: (1) The SK and BK components of Ca^2+^-activated K^+^ currents present in SPNs (Pineda et al., 1992; Bargas et al., 1999; Vilchis et al., 2000), participate in shaping the corticostriatal polysynaptic responses. Basically, these currents participate in synaptic integration controlling the duration and depolarization of the responses. (2) The role of Ca^2+^-activated K^+^-currents is different in dSPNs as compared to iSPNs: in dSPNs, Ca^2+^-activated K^+^ currents produce slow gradual responses, suggesting that a diffusion delay occurs between Ca-entry and K^+^-currents activation. In contrast, Ca^2+^-activated K^+^-current action measured in the same way in iSPNs is fast rising and thereafter maintained during a large portion of the response, suggesting that they are activated immediately at the beginning of the synaptic inputs. In fact, Ca^2+^-activated K^+^ currents appeared to regulate small subthreshold synaptic potentials in iSPNs (Higley and Sabatini, 2010) and much less so in dSPNs. (3) We show that Ca^2+^-activated K^+^-currents are main determinants of the time courses of the corticostriatal responses in SPNs: enhancement of these currents accelerated the repolarization of dSPNs making them to look as iSPNs. Correspondingly, their blockade prolongs the corticostriatal responses in iSPNs making them to look as dSPNs. Finally, (4) a main role is played by Ca^2+^-activated K^+^ currents in iSPNs. It is known that when these neurons are activated, they may elicit regenerative events, seen as all-or-none events that, when propagation ensues, can become full-blown calcium action potentials as recorded from the soma. When this occurs, the burst of action potentials that should go out to the axon is obliterated.

### Outputs of dSPNs and iSPNs have different durations

Before BAC-mice were available, investigators averaged the output of SPNs thinking that different durations were a part of the same spectrum. However, BAC-D_1/2_ GFP mice have taught us that iSPNs and dSPNs have different excitable properties (Shen et al., 2007; Day et al., 2008; Kravitz et al., 2010; Gerfen and Surmeier, 2011), and that their responses to intracortical stimulation are differentially integrated (Flores-Barrera et al., 2010), even though responses to stimulation at the dendrites, with uncaged glutamate, and the glutamate receptors employed, do not seem different (Plotkin et al., 2011; Vizcarra-Chacon et al., 2013). However, once SPNs classes were sorted out, differences in duration of their physiological responses—trains of action potentials—were easily observed, even during extracellular recordings under certain conditions (optogenetic stimulation; e.g., see Figures 2E–H in: Kravitz et al., 2010). How to explain these differences in duration? In the present work we show that Ca^2+^-activated K^+^-currents are a main factor in explaining the duration of corticostriatal responses.

Apparently, dendritic Ca^2+^-activated K^+^-currents limit calcium influx and depolarization, making a negative feedback loop (Faber, 2009). Since a stronger calcium entry has been previously reported in iSPNs (Day et al., 2008), the functional importance of Ca^2+^-activated K^+^-currents in determining the threshold and duration of dendritic autoregenerative potentials (most probably due to Ca^2+^: Bargas et al., 1991; Carter and Sabatini, 2004) is fundamental to avoid their propagation in order to be able to generate the brief train of action potentials that goes out to the axon. The action of these currents at subthreshold levels shows that the calcium to activate them is available even with weak synaptic stimuli (Higley and Sabatini, 2010). However, at the level of dendritic spines, blockage of some Ca^2+^ sources could have paradoxical effects increasing the amplitude of synaptic events (Higley and Sabatini, 2010). But at suprathreshold levels, where up-states could arise, NMDA receptors, and SK channels are functionally coupled in the dendritic spines of hippocampal, amygdala, and striatal neurons (Bloodgood and Sabatini, 2005; Ngo-Anh et al., 2005; Lujan et al., 2009; Faber, 2010; Higley and Sabatini, 2010) to control the duration and depolarizing level of plateau potentials (Cai et al., 2004), limiting the influx of calcium through NMDA receptors and controlling the amount of excitation (Bond et al., 2005; Ngo-Anh et al., 2005; Faber, 2010; Tonini et al., 2013).

But why the activation of Ca^2+^-dependent K^+^-currents is faster in iSPNs than in dSPNs so that repolarization of responses in iSPNs makes them much briefer? Apparently, eliciting local autoregenerative responses in the dendrites, at the time of synaptic inputs, is more common in iSPNs than in dSPNs (Flores-Barrera et al., 2010; Higley and Sabatini, 2010). A brisk Ca^2+^ entry may explain the fast rise in the activation and actions of Ca^2+^-activated K^+^-currents, and therefore the faster repolarization of iSPNs after a brief burst of action potentials.

However, these are not the only intrinsic currents that participate in shaping the suprathreshold corticostriatal response or in eliciting autoregenerative responses in SPNs. Other inward currents participate. As for example, the slow inward current carried by Ca_V_1 channels (Galarraga et al., 1997; Vergara et al., 2003; Carter and Sabatini, 2004; Flores-Barrera et al., 2011), a slow sodium component blocked by phenytoin and riluzole (Carrillo-Reid et al., 2009b), inward currents carried by Ca_V_3 and Ca_V_2.3 channels (Carter and Sabatini, 2004; Higley and Sabatini, 2010; Plotkin et al., 2011), all them besides the synaptically activated NMDA and kainate slow current components (Schiller and Schiller, 2001; Carter and Sabatini, 2004; Plotkin et al., 2011; Vizcarra-Chacon et al., 2013). But analyses have not been complete, other inward currents need to be confirmed or discarded in mature neurons as for example currents carried by TRP channels (Hill et al., 2006) or calcium-activated non-selective cation currents (ICAN; Mrejeru et al., 2011).

Further research is needed to see if different synapses (proximal, distal) use the same calcium source, or if there could be many sources being either the same or diverse for different synapses. It is known that voltage-gated Ca^2+^-channels and NMDA receptors differ in their distribution over spines and dendritic shafts. High calcium concentrations can only be reached within a few nanometers around the channel and dissipate within microseconds (Sabatini et al., 2002). So that the Ca^2+^-source used to activate Ca^2+^-dependent K^+^-currents may be very specific and precise (Vilchis et al., 2000). In contrast, NMDA-channels lead to a longer lasting calcium gradient extending across the spine during an excitatory postsynaptic potential (Sabatini and Svoboda, 2000; Keller et al., 2008). In any case, the Ca^2+^ source should be able to generate autoregenerative events to explain the differences in duration of the responses between dSPNs and iSPNs.

In view of the above, it should not seem strange that intrinsic outward currents should also participate in suprathreshold corticostriatal responses to damp or stop inward currents from reaching too strong depolarizations. In the present work we show that Ca^2+^-activated K^+^-currents are a component of the outward currents that fulfill this role. They have been described at the somatic (Bargas et al., 1999) and the dendritic levels (Ngo-Anh et al., 2005). However, several other outward currents may participate together with the synaptic GABAergic currents (Flores-Barrera et al., 2010) in order to control suprathreshold responses originated by converging polysynaptic cortical inputs known to outlast the initial stimulus and the monosynaptic responses by several hundreds of milliseconds (Vizcarra-Chacon et al., 2013).

It is known that these prolonged responses arise from the polysynaptic convergence of cortical and feed-forward striatal inputs because in current-clamp mode the glutamatergic response can clearly be divided into two parts, an early part, which is monosynaptic, and a late part, which by definition cannot be monosynaptic, but is blocked by AMPA-receptor antagonists (Vizcarra-Chacon et al., 2013), that is, it has to be polysynaptic. In addition, in voltage-clamp mode, at certain holding potentials, a late barrage of synaptic inputs keeps arriving during several hundreds of milliseconds after the initial monosynaptic response (Goldberg et al., 2012; Vizcarra-Chacon et al., 2013). Either stimulus strength or holding potential have to be changed for the activation of unclamped intrinsic currents by this synaptic barrage (Flores-Barrera et al., 2009). Finally, several striatal interneurons can be activated from the cortex (e.g., Vizcarra-Chacon et al., 2013) and the feed-forward circuit that involves other striatal cellular elements in the suprathreshold corticostriatal response of SPNs is well known (rev. in: Surmeier et al., 2011).

## Conclusion

Ca^2+^-activated K^+^ channels, most probably located at the dendrites, explain the different durations between corticostriatal responses in dSPNs and iSPNs. Because polysynaptic corticostriatal responses (Vizcarra-Chacon et al., 2013) are the components of more prolonged up-states (Stern et al., 1997), an inference can be made: transmitters that modulate Ca^2+^-influx or Ca^2+^-activated K^+^-currents intervene in the regulation of up-states duration. This in turn regulates correlated firing among neuronal ensembles to produce network dynamics (Carrilo-Reid et al., 2008). Therefore, they may be a part of the explanation of the actions of modulatory transmitters at the striatal microcircuit (Carrillo-Reid et al., 2009a; Jáidar et al., 2010; Carrillo-Reid et al., 2011).

### Conflict of interest statement

The authors declare that the research was conducted in the absence of any commercial or financial relationships that could be construed as a potential conflict of interest.
